# TRIO Platform: A Novel Low Profile *In vivo* Imaging Support and Restraint System for Mice

**DOI:** 10.3389/fnins.2016.00169

**Published:** 2016-04-25

**Authors:** Vladislav Voziyanov, Benjamin S. Kemp, Chelsea A. Dressel, Kayla Ponder, Teresa A. Murray

**Affiliations:** ^1^Integrated Neuroscience and Imaging Lab, Center for Biomedical Engineering and Rehabilitation Sciences, Louisiana Tech UniversityRuston, LA, USA; ^2^School of Biological and Health Systems Engineering, Ira A. Fulton School of Engineering, Arizona State UniversityTempe, AZ, USA

**Keywords:** *in vivo* imaging, head restraint, cranial window, anesthesia, warming pad, gradient index lens, multiphoton microscopy, astrocytes

## Abstract

High resolution, *in vivo* optical imaging of the mouse brain over time often requires anesthesia, which necessitates maintaining the animal's body temperature and level of anesthesia, as well as securing the head in an optimal, stable position. Controlling each parameter usually requires using multiple systems. Assembling multiple components into the small space on a standard microscope stage can be difficult and some commercially available parts simply do not fit. Furthermore, it is time-consuming to position an animal in the identical position over multiple imaging sessions for longitudinal studies. This is especially true when using an implanted gradient index (GRIN) lens for deep brain imaging. The multiphoton laser beam must be parallel with the shaft of the lens because even a slight tilt of the lens can degrade image quality. In response to these challenges, we have designed a compact, integrated *in vivo* imaging support system to overcome the problems created by using separate systems during optical imaging in mice. It is a single platform that provides (1) sturdy head fixation, (2) an integrated gas anesthesia mask, and (3) safe warm water heating. This THREE-IN-ONE (TRIO) Platform has a small footprint and a low profile that positions a mouse's head only 20 mm above the microscope stage. This height is about one half to one third the height of most commercially available immobilization devices. We have successfully employed this system, using isoflurane in over 40 imaging sessions with an average of 2 h per session with no leaks or other malfunctions. Due to its smaller size, the TRIO Platform can be used with a wider range of upright microscopes and stages. Most of the components were designed in SOLIDWORKS® and fabricated using a 3D printer. This additive manufacturing approach also readily permits size modifications for creating systems for other small animals.

## Introduction

Laser scanning confocal microscopy through cortical windows has opened upper layers of the mouse brain for high resolution, time-course, *in vivo* imaging (Didion et al., 2005; Brown et al., 2010) and multiphoton microscopy (MPM) has facilitated *in vivo* imaging of the olfactory bulb (Adam and Mizrahi, 2011) and lower layers of the cortex, up to several 100 microns deep (Levene et al., 2004). Additionally, implanted gradient index (GRIN) lenses have extended the reach of MPM to lower cortical and subcortical regions of the murine brain (Levene et al., 2004; Barretto et al., 2011; Murray and Levene, 2012) and small prisms have provided vertical views of entire cortical columns (Chia and Levene, 2009). These technological achievements have met at a crossroad with the rapid growth in the number of transgenic mouse strains to achieve unprecedented spatiotemporal resolution of dynamic processes in the brain. Transgenic mice with fluorescent proteins expressed under specific genetic promoters have permitted cell-type specific identification of targeted cell populations. By using these mice for *in vivo* imaging, investigators have observed processes such as changes in dendritic spine density (Stetter et al., 2013), migration and activation of microglia (Nimmerjahn et al., 2005), growth of brain tumors (Brown et al., 2010; Barretto et al., 2011) and the dynamics of a brain tumor microenvironment (Ricard and Debarbieux, 2014). Additionally, genetically encoded calcium sensitive fluorescent proteins (Hoogland et al., 2009; Barnstedt et al., 2015) and dyes (Svoboda et al., 1997; Hoogland et al., 2009) have revealed the activity of local and large scale neuronal networks (Dombeck et al., 2007). Furthermore, by counterstaining the vasculature, blood flow rates have been determined (Pinard et al., 2000; Levene et al., 2004), and vessel permeability and leukocyte trafficking have been observed (Pai et al., 2012). The ability to monitor these activities over time in a mouse model has tremendous importance to both basic research and preclinical studies (Ricard and Debarbieux, 2014; Lee et al., 2016). Yet, the means by which mice are positioned and maintained for imaging has relied primarily on old technology and non-standardized custom made components.

Positioning and maintaining anesthetized mice for imaging over time requires a means to secure the head, maintain the animal's body temperature and facilitate delivery of anesthesia. Securely restraining the animal's head during imaging is essential for reducing motion artifacts from respiration and heart beats (Pai et al., 2012). A good restraint system will also optimize the alignment of the imaging region with the microscope objective (Murray and Levene, 2012; Pai et al., 2012). Repeatable alignment is important for longitudinal experiments and is critically important for imaging through implanted GRIN lenses (Gilsdorf and Palais, 1994).

Numerous methods of restraining mice via head fixation for *in vivo* imaging have been reported. These mainly include stereotaxic devices (Ricard and Debarbieux, 2014) many of which are custom fabricated for a particular research lab (Brown et al., 2010; Adam and Mizrahi, 2011; Pai et al., 2012), custom designed stages (Barnstedt et al., 2015), and custom-made bars or plates (head posts) permanently affixed to the head with customized stage-mounted devices for securing the head post (Shih et al., 2012; Stetter et al., 2013; Goldey et al., 2014; Barnstedt et al., 2015). Several commercially available head immobilization devices are available that can be used for restraining the head for imaging through cranial windows (MAG-1 Simple Head Holder Plate for Mice from Narishige International USA, Inc. and mouse and neonatal rat adaptors from Stoelting Co. and Harvard Apparatus, Harvard Biosciences, Inc.). However, these are 4–7.5 cm tall, which is greater than the distance between the stage and the objective lens on some microscope systems. Three-point immobilization devices with ear bars and a bite bar are smaller than stereotaxic frames and will securely immobilize the head, but these have rudimentary adjustments for pitch, yaw, and roll. This makes it difficult to achieve the same alignment over multiple imaging sessions for longitudinal studies. Some custom head plate and holder systems have a much smaller size and consistent alignment for imaging through cranial windows. Unfortunately, recreating custom components can pose a challenge when attempting to use another lab's protocol. Notably, no low profile, commercially available systems were identified for achieving the repeatable, sensitive alignment needed for repeated imaging through an implanted GRIN lens.

Gas anesthesia may be used for long *in vivo* imaging sessions to avoid unwanted potential movement that can result from using injectable anesthetic agents and to avoid light contamination when opening a shroud to make an injection. However, the nose cones for gas anesthesia are usually configured to mount onto stereotaxic frames and very few papers report using stereotaxic frames for *in vivo* imaging (Brown et al., 2010; Ricard and Debarbieux, 2014). Presumably, this is because of their large size. Alternatively, a simple plastic cone can be attached to the hose from the vaporizer. The open end of the cone is covered with the top half of a rubber balloon with a slit in it. The nose of the mouse is placed in the slit of the cone, but if the slit is not properly sized, anesthetic gas can leak into the room (personal observation) unless a scavenger system is also installed. This increases the amount of space occupied by components on and around the microscope stage and can add substantial amount of set up time.

Prolonged anesthesia will cause a drop in body temperature to hypothermic levels if left unchecked (Caro et al., 2013). Two commonly used types of systems are available. These are electrical, thermostatically controlled resistive heating plates or pads and warm water pads. Electrical plates are thin and fit well under microscope objectives. Both the electrical plates and pads pose a risk of overheating the animal if the temperature probe is improperly placed or it becomes dislodged. Warm water pads use circulating water that is warmed in a reservoir to a set temperature, such as 37°C. Its thermostat is located inside the water tank, which greatly reduces the possibility of overheating the animal. Unfortunately, these pads are bulky and raise the height of the mouse by several millimeters or more. They can also be uneven and thus take additional time moving the pad to achieve proper head alignment. To our knowledge, none of these heating systems provide head fixation. Using a heating pad with a standard immobilization frame raises the height of the mouse's head to over 7 cm above the microscope stage.

## Materials and methods

### TRIO platform design and construction

**Figure 1:**
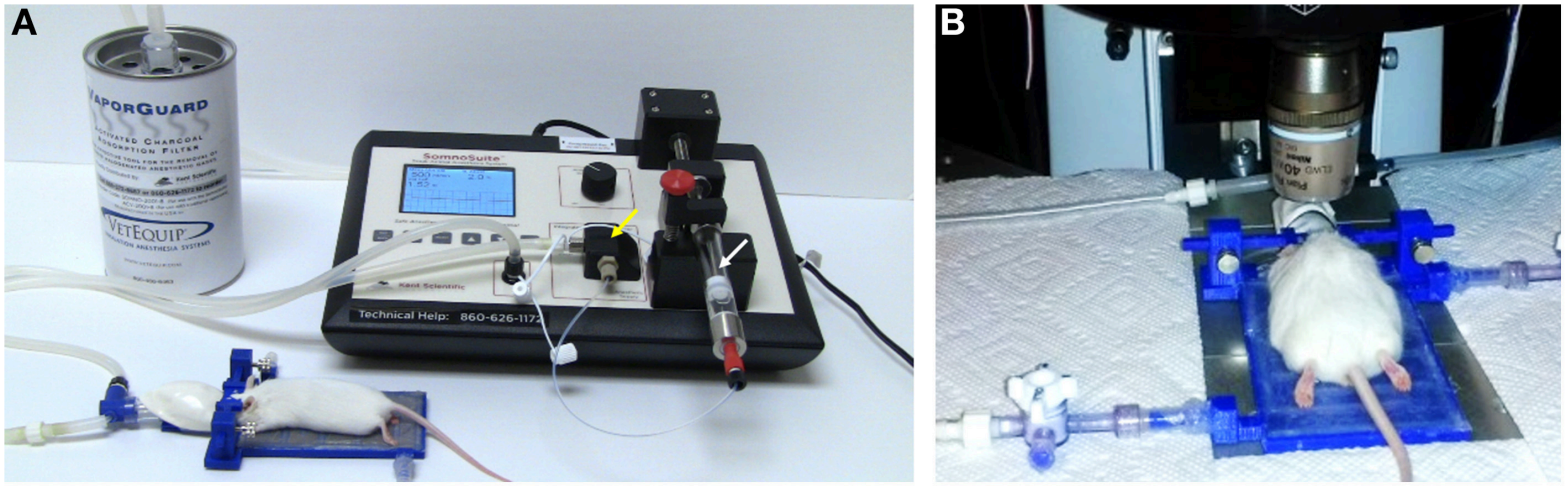
**TRIO Platform: an *in vivo* integrated imaging support system**. **(A)** TRIO Platform with gas anesthesia supplied by a compact, portable gas anesthesia system (SomnoSuite®, Kent Scientific Corp.). Isoflurane is supplied from a syringe (white arrow). The integrated digital vaporizer (yellow arrow) is much smaller than traditional vaporizers which markedly reduces the amount of anesthesia used for surgeries and for imaging sessions. **(B)** Anesthetized mouse positioned and warmed for imaging using the TRIO Platform on the stage of a multiphoton microscope. The overall height of the base plate with circulating warm water and the mouse head is only 20 mm. This relatively small height will enable its use on a wider range of microscopes (The white paper towels around the platform were used to block reflections from the lights used for this photo. This is not required for imaging). The compact anesthesia system can be placed to the side of the stage within the microscope enclosure or outside of the enclosure.

**Figure 2:**
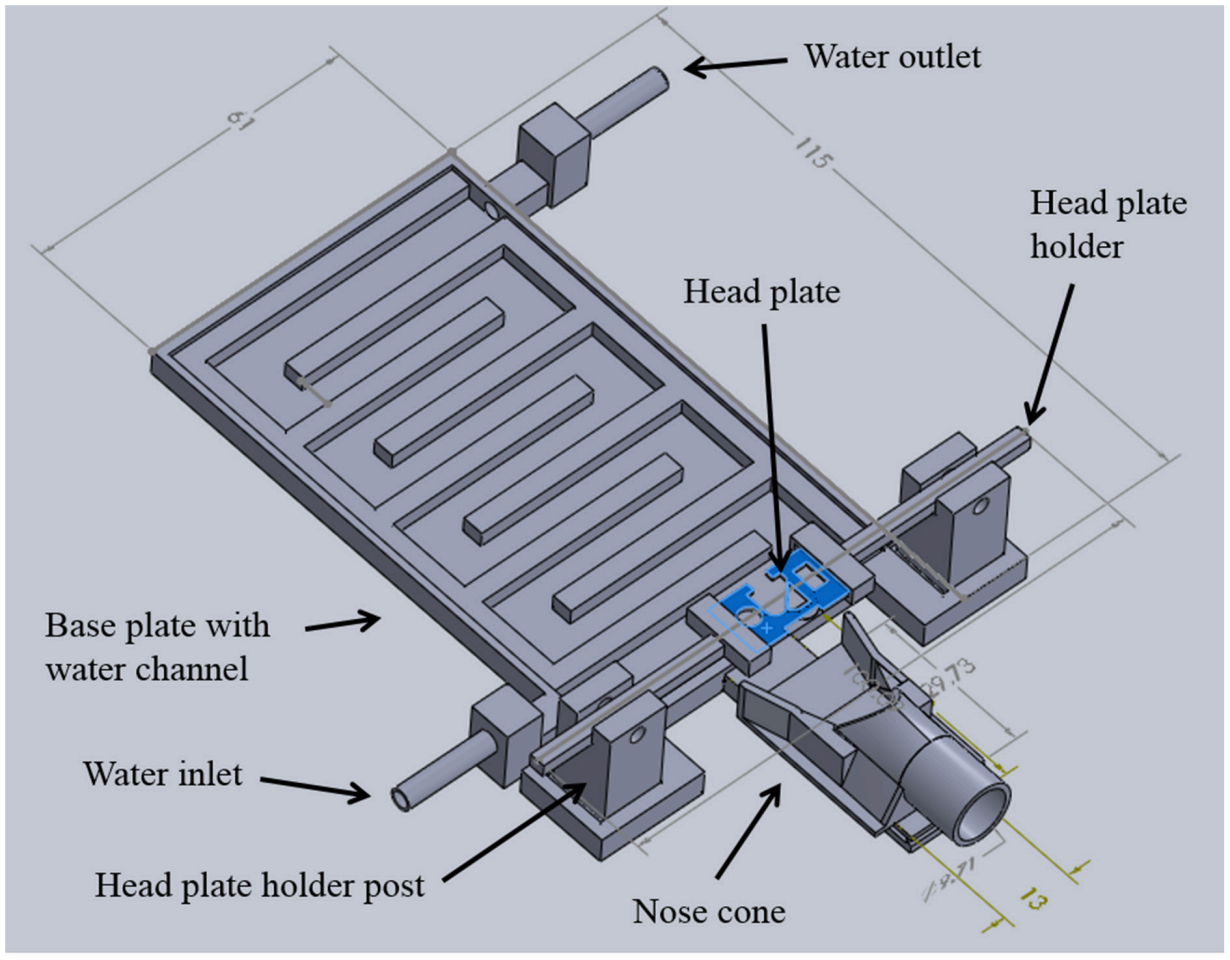
**TRIO Platform design**. Integrated system with the top cover of the base plate removed to show the serpentine water channel. 3D printed parts were designed in SolidWorks®. Dimensions are in mm (See Supplementary Material for design details.)

The system was designed in order to restrain the head of an anesthetized mouse with a head plate (or head post) attached to the skull and to orient the cranial window so that the flat face of the window is perpendicular to a microscope objective (Figure 1). The system was also designed to provide gas anesthesia and to maintain body temperature. The components of the system were designed in SolidWorks (Dassault Systèmes; Figure 2). A MakerBot® Replicator® 2 three-dimensional (3D) printer and poly (lactic acid) (PLA) filament were used to fabricate the system. This manufacturing method minimized development time and costs, allowing for multiple design iterations. The widespread availability of 3D printers makes it possible for other researchers to replicate or modify the design as their experiments require. Detailed descriptions of the TRIO system components are provided below. The SolidWorks(R) files for printing the components and a list of non-printed parts are available online in the Supplementary Materials Section in Design Files S1 and Table S2, respectively.

### Warm water plate

A 3D printed plate with an interior, serpentine channel for warm water circulation serves as the central hub of the system (Figure 2). The plate was designed with a low-profile rectangular geometry that is 5 mm high, 115 mm long, and 61 mm wide. Water inlet and outlet ports were placed at opposing corners and fitted with standard tube fittings to allow attachment of polymer tubing to deliver warm water and recycle it back to the warm water reservoir. Water in the reservoir was maintained at 40°C. A flat cover was attached to the top of the water chamber with cyanoacrylate adhesive (Loctite). The entire chamber was sealed with a silicone adhesive (Kwik Sil, World Precision Instruments, Sarasota, FL, USA) to prevent leaks. This system has been used for 9 months without any water leaks.

### Anesthesia nose cone

**Figure 3:**
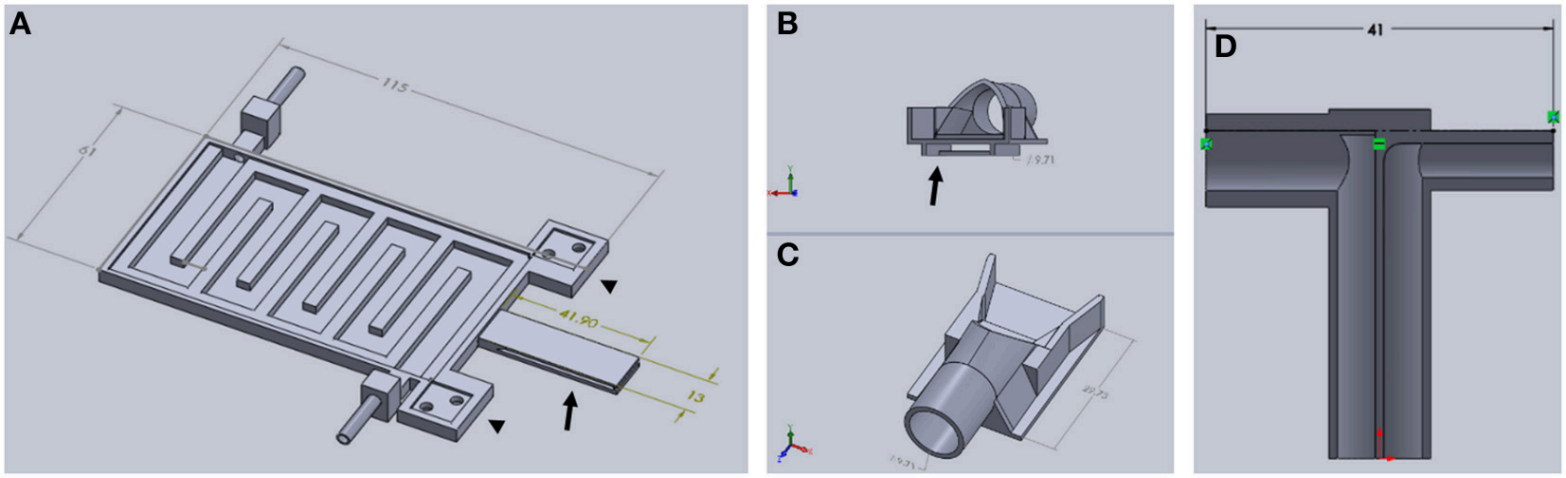
**Components of TRIO Platform**. **(A)** This view shows features of the base plate that cannot be seen in Figure 2 above, such as the extensions of the base plate for attaching the head plate holder posts (arrowheads) and the slotted tongue (arrow) for attaching the nose cone. **(B,C)** Two views of nose cone design. Channel under nose cone in (**B**, arrow) slides into a slot in the tongue (**A**, arrow). **(D)** Gas inlet (right side) and outlet (left side) are integrated into one piece that attaches to the tubular component of the nose cone **(C)** (Dimensions are in mm).

To deliver a gaseous anesthetic, a slotted tongue (Figure 3A, arrow) was glued to the front of the printed plate and a 3D printed nose cone (Figures 3B,C). The channel under the nose cone (Figure 3B, arrow) slides into the tongue's slot and is held in place by a triangular catch, preventing it from being dislodged accidently. The cone can be removed for cleaning or when using an alternative anesthetic.

**Figure 4:**
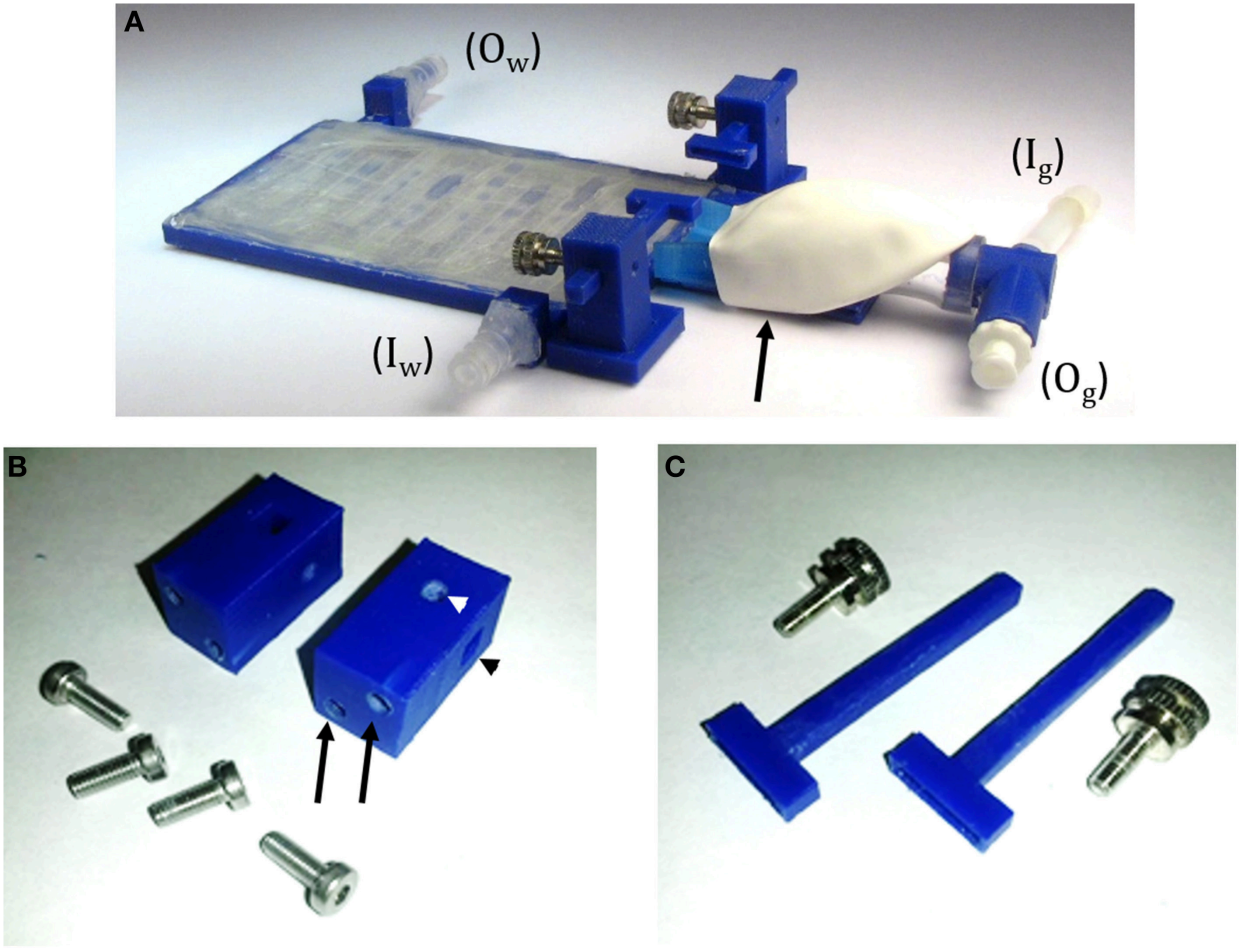
**TRIO Platform 3D-printed system. (A)** Assembled system with tube fittings for attachment to inlet (I_w_) and outlet (O_w_) water lines and Luer lock fittings for attachment of inlet (I_g_) and outlet (O_g_) tubes from a gas anesthesia system. The elastomeric shroud is held onto the nose cone with plastic tape from the underside of the device (black arrow). **(B)** Head plate holder posts and machine screws. Two screws are placed in holes in the base plate and screwed into tapped holes in the posts (black arrows) to attach the posts to the base plate. A square hole in the post holds an arm of the head plate holder (black arrowhead) and a round taped hole in the post (white arrowhead) holds a set screw **(C)** to secure the position of the arm in the square hole. **(C)** Head plate holders with the accompanying set screws that secure the head plate holders to the holder posts.

A t-shaped gas delivery fitting, also printed with PLA, (Figure 3D) is connected to an opening in the cone (Figure 3C, arrow). One side has a tube fitting that connects to the inlet hose from a compact gas anesthesia system. The other side connects to the hose that returns gas to the anesthesia system where the scavenged gas is trapped in an activated charcoal filter. Both hoses are made of silicone tubing (FDA-grade, Platinum-cured silicone tubing, 1/8″ ID × 3/16″ OD, VWR International). Due to its extraordinarily small size, we used a SomnoSuite™ anesthesia system (Kent Scientific Corp.) because it fits easily within or near the housing that surrounds our multiphoton microscope, which was a Vivo™ 2-Photon Microscopy Workstation (Intelligent Imaging Innovations, Inc., also written as 3i, Inc.). In order to make isoflurane delivery more efficient and minimize leaks of anesthetic into the workspace, an elastomer shroud (AC-COAX-Balloon, Kent Scientific Corp.) is secured over the opening of the nose cone with a piece of 1/2-wide plastic tape (Figure 4 arrow). The elastomer covering can be lifted temporarily to confirm that the animal's nose is place fully in the cone. A medium-sized rubber balloon with the neck piece removed will work equally as well. We have also used a piece of 18-gauge wire to secure the shroud to the gas delivery tube instead of tape. The shroud was easily replaced when the edges of the rubber dried and cracked after several months of use.

In order to add a head plate holder securely to the base plate, the two front corners of the base plate have two 21 mm square extensions that are printed as part of the bottom plate (Figure 3A, arrowheads). The posts are attached to these extensions using 3-mm hex head machine screws 8 mm in length. The posts are rectangular columns, 22 mm in height. Each post has two 8-mm long holes in the bottom (Figure 4B, black arrows) that are tapped to match the thread pitch of the 3-mm hex head machine screws that hold the post to the base plate. Each post also has a square hole (4 × 4 mm, black arrowhead) for holding the long arm of a head plate holder (Figure 4C), and one 3-mm circular hole (white arrowhead) that is tapped to match the thread pitch of a 3-mm set screw with a knurled head for hand tightening. The long arm of each of the two head plate holders has a square profile that fits snugly into the square holes in the holder posts. The arm can be securely positioned in the post by tightening the set screw by hand until the arm does not move with gentle pressure (do not overtighten the screw as this will cut into the plastic and shorten the life of the holder). The t-shaped end of each bar has a 1-mm high by 1-mm deep rectangular cavity that is 12 mm long (Figure 3, inset), which holds the outer end of a head plate. Together, both bars securely hold the head plate and orient it such that the cranial window is perpendicular to the laser beam.

### Head plate and head plate assembly

SolidWorks and 3D printing with a PLA filament (G-Star Technologies) were used to design and fabricate head plates, respectively. We used a polymer head plate instead of a metal plate or bar to minimize the mass of the plate and permit easy modification of the number of ports and their placement. Minimizing the mass of the head plate, which was ~0.2 g, was important for our work because many of our mice are used for behavioral experiments in between imaging sessions (Lee et al., 2016).

**Figure 5:**
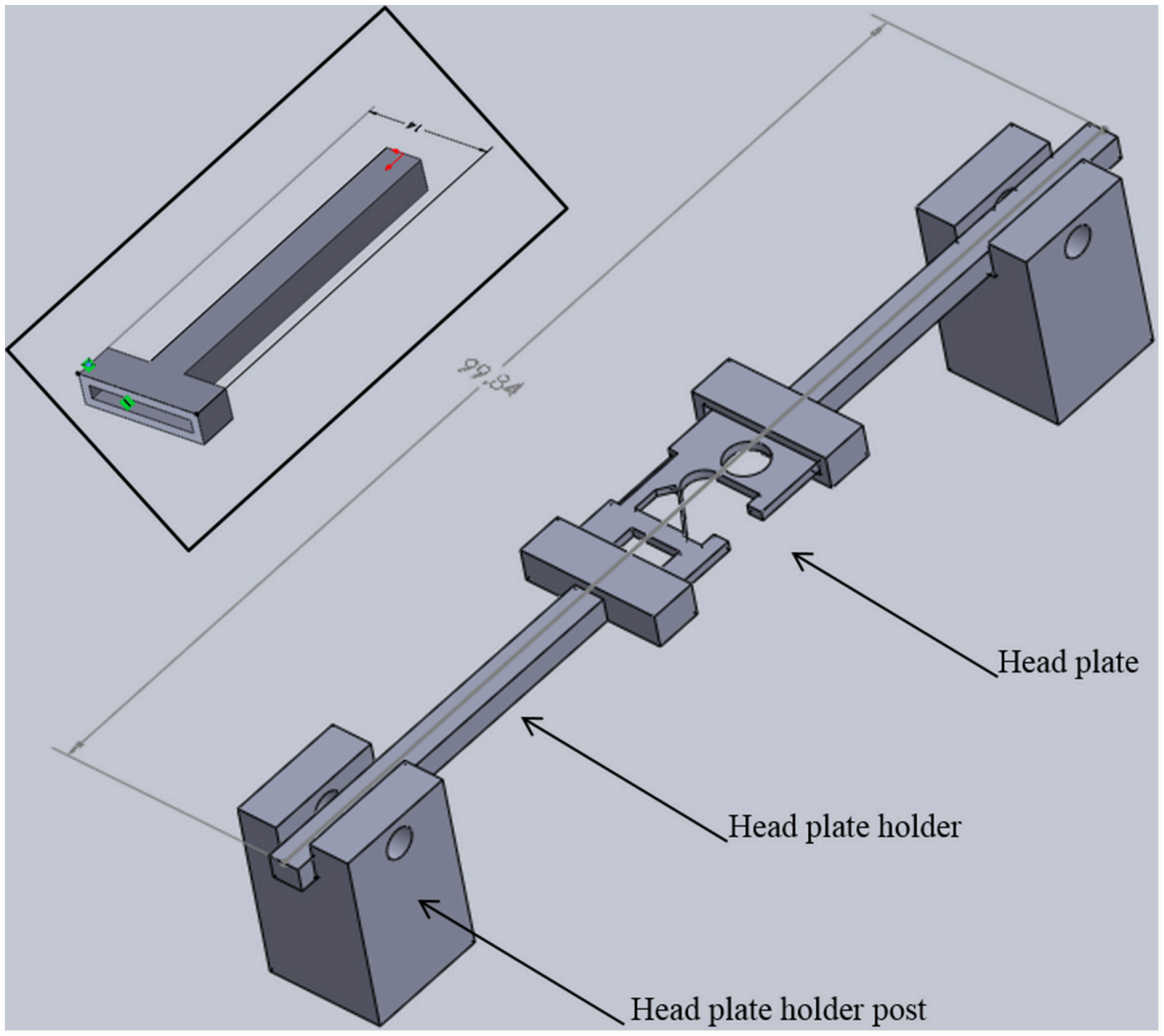
**Head plate holder assembly**. Two head plate holders grip each side of a head plate. The head plate is permanently attached to the skull of a mouse. Head plate holder posts position the holders and head plate above the base plate with sufficient space for positioning the head of an adult mouse (see Figure 1B). The entire assembly is 22 mm tall, 14 mm wide, and just under 100 mm long. **Inset:** Detailed view of a head plate holder. The long arm has a square cross section (3.7 × 3.7 mm) that fits into a square hole in a holder post. One end of the head plate fits into the slot in the t-shaped end. The slot is 12 mm long, 1 mm wide, and 1 mm deep. The overall width is 14 mm and the overall length is 41 mm. These dimensions can be adjusted for different sized head plates.

**Figure 6:**
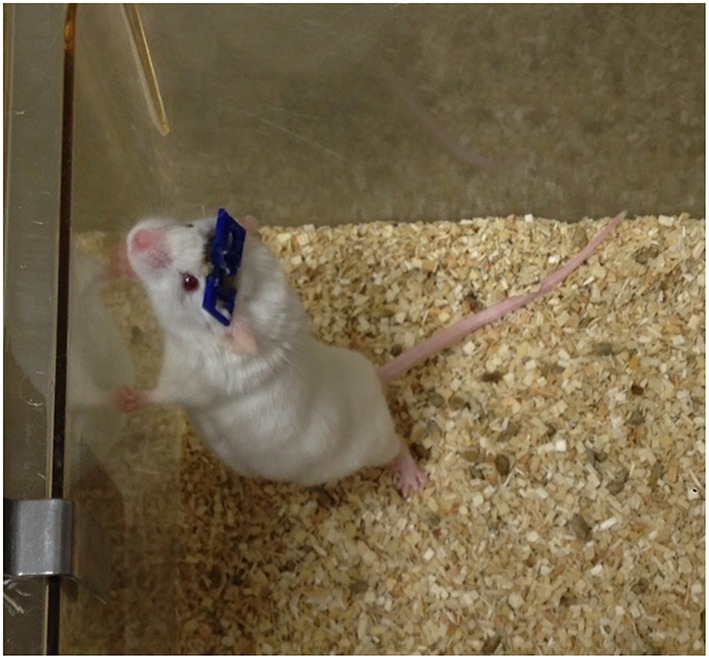
**Mouse with an implanted GRIN lens and permanently attached head plate**. Prior to surgery, a GRIN lens was affixed to a 5-mm diameter cover slip which was then glued to the head plate. The lens was implanted into the brain by lowering it through a craniotomy and advancing it into the brain as the head plate was slowly lowered to the skull (see Figure 7).

The head plates are 9 mm long, which is long enough to have free ends to attach to the head plate holders (Figures 2, 5) and short enough to provide free movement when the mouse is awake (Figure 6). A section at the caudal edge is cut out, providing an opening for installation of an injury hub which was used for midline fluid percussion experiments to induce traumatic brain injury (Lifshitz et al., 2007). If the hub is not needed, the cutout provides additional surface area for applying adhesive. A second cut out (Figure 5), provides a place to attach a 5-mm diameter glass cover slip cranial window.

Optical adhesive (Number 71 Adhesive, Norland Products, Cranbury, NJ, USA) is used to secure the cover slip to the head plate. A thin bead of adhesive (~1-mm wide) is applied around the edge of the cover slip and then gently pressed against the bottom of the head plate using blunt, curved forceps (place head plate upside down for this step) so that the adhesive forms a continuous seal. The optical adhesive is cured using a UV lamp according to the manufacturer's instructions (Safety note: UV light is harmful. Shield eyes and skin from UV light at all times). It is important to confirm that the coverslip is parallel to the bottom of the head plate which will ensure optimal alignment of the cranial window for imaging. If the resolution of the printer is not precise enough, the cover slip may not seat parallel to the head plate. In this case, the area for attachment can be milled using a 5-mm end mill and standard machining techniques.

For deep brain imaging in lower cortical layers 4–6 and subcortical regions, a GRIN lens should be attached to the cranial window (5-mm diameter glass cover slip). The attachment of the GRIN lens to a cover slip has been described in our previous work (Murray and Levene, 2012). Because the cover slip is mounted on the underside of the head plate, the head plate should be placed upside down and the coverslip-GRIN lens assembly should be positioned over an appropriate opening in the head plate with the free end of the GRIN lens pointed up. It is recommended that this procedure be done using a stereomicroscope or large magnifying glass with ample lighting to clearly visualize the forceps and lens and to ensure that the forceps do not touch the GRIN lens. The forceps can transfer glue and debris onto the lens which could degrade image quality. It is also possible to chip the edge of the lens or to break it off the cover slip with sufficient pressure. In our experience, workers become proficient at making these assemblies in about 2–3 procedures.

It is important to create a clean, dust-free or low-dust work area for attaching the cover slip to the head plate. This is to reduce potential sources of infection and to maintain optical clarity of the window and/or GRIN lens. Components can be cleaned with 70% ethanol prior to assembly to remove any dust particles before applying adhesives. Additionally, the UV light used for curing the adhesive disinfects the exposed surfaces. Assemblies should be transferred after curing using clean forceps and stored in clean, covered containers. Prior to implantation, assembled head plates should be exposed to UV light for 15 min on each side or soaked in 70% ethanol for 15 min to disinfect the surfaces. They should be transferred to the surgical site in sterile containers. Autoclaving is not recommended as it might warp the polymer head plate. Ethylene oxide gas was not tested as a means of sterilization.

Because the head plate design is easily modified using SolidWorks® and plates are quickly printed with an inexpensive polymer, making changes for different types of imaging experiments is quick and economical.

### Printing the TRIO platform components

All polymer parts were designed in SolidWorks® and produced using a Replicator 2® (MakerBot®) 3D printer. All pieces were printed at 100% fill with a 0.1 mm layer height (Note: Some 3D printers cannot print as small as 0.1 mm/layer). Optimal 3D printing of the components of this system requires correct printing orientation. Printing in another orientation could result in the printer program adding a meshwork of polymer “supports” to overhanging areas. Without supports, overhanging areas would deform under the force of gravity. These supports could occlude channels and slots that should be left open. It is possible to orient the parts with open areas so that supports are not needed. We have optimized the orientations for printing each part, as follows:

1. The base of the platform was printed right side up, with the bottom of the device flat against the platform, with no supports.
2. The top of the device was printed on its widest side (i.e., flat) with no supports.
3. The slotted tongue was printed on its thinnest side so that the slot was perpendicular to the printer platform. No supports were used.
4. The nose cone was printed with the cylindrical part against the platform with supports.
5. The gas delivery fitting was printed in two halves, with the inside edges flat against the platform, with no supports.
6. The head plate holder post was printed laying on its longest side, so that the slot was perpendicular to the platform, and with no supports.
7. The head plate holder was positioned so that the long arm was pointed straight into the air and printed with no supports.

### Animals used for *in vivo* imaging tests

White, GFAP-GFP^+^ lab mice (FVB/N-Tg(GFAPGFP)14Mes/J) were obtained from Jackson Labs. Louisiana Tech University IACUC guidelines were followed for animal housing with lighting in a 12 h on–12 h off cycle. Food and water were provided *ad libitum*.

### Surgery

All surgical and imaging procedures were approved by the Louisiana Tech University Institutional Animal Care and Use Committee. Every effort was made to minimize pain and suffering. A noxious stimulus consisting of two toe pinches every 15 min was used to confirm that animals were in a surgical plane of anesthesia during surgical procedures. Mice were monitored for recovery until sternal recumbency was observed. To minimize post-surgical pain, Ibuprophen, children's suspension (30 mg/kg/day) was dissolved in the animal's drinking water for 60–72 h following surgery. Mice were also monitored daily for signs of distress or poor health. The end point for mice was perfusion (see histology section).

Prior to performing any procedures, mice were allowed to acclimate to the room in their home cage for at least 30 min. For surgery, a mouse was initially anesthetized with 3% isoflurane (VEDCO) in the induction chamber of a SomnoSuite® small animal anesthesia system (Kent Scientific Corp.). A mixture of ketamine (10 mg/kg) and xylazine (1 mg/kg) in sterile saline was injected intraperitoneally after induction of anesthesia using the isoflurane chamber. This was done to give surgical staff time to prep the animal and position it in the stereotaxic frame. Once secured in the frame, the mice were maintained on 1.5% isoflurane. Ophthalmic ointment was placed on the eyes and the fur over the scalp was cut using iris scissors. After this, the mouse was placed in a Benchmark stereotaxic frame with a nose cone for gas anesthesia (Leica Biosystems) and a mouse and neonatal rat adaptor (Stoelting Co.). Anesthesia was maintained using 1.5% isoflurane supplied by the SomnoSuite® system. Body temperature was regulated and heart rate and SpO2 were monitored using a PhysioSuite® System (Kent Scientific Corp.).

Aseptic surgery was performed to install either a cranial window or a GRIN lens mounted on a cranial window, as previously described (Murray and Levene, 2012; Lee et al., 2016). Briefly, after confirming the mouse was in a surgical plane of anesthesia using noxious toe pinches, an incision was made in the scalp and connective tissue was cleared.

Under a surgical microscope, a micro drill was used to make a 1-mm diameter craniectomy at selected rostral and lateral distances with respect to bregma and the midline, respectively. The exposed dura was removed and a GRIN lens (mounted on a head plate, as described in the head plate assembly section) was implanted through the craniectomy.

**Figure 7:**
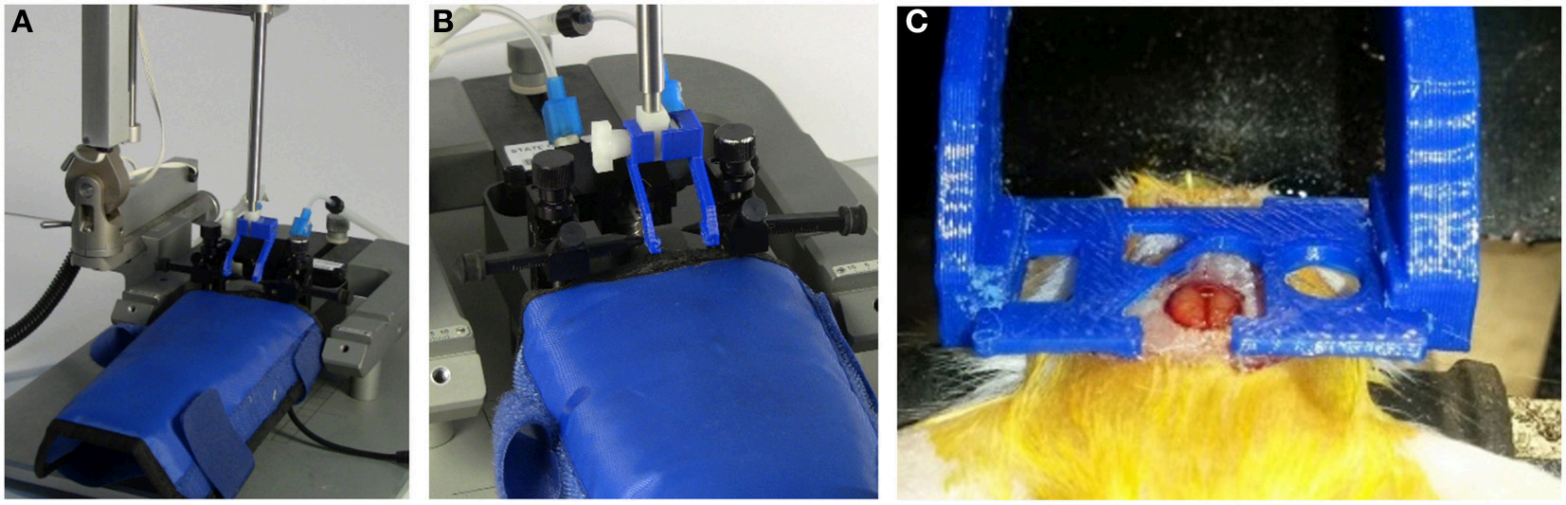
**Probe holder with custom grips for surgically attaching head plate**. The head plate is held by 3D printed head plate grips that are attached to the end of a standard probe holder for a stereotaxic device (see Supplementary Material). **(A)** Probe holder with custom grips attached. **(B)** View showing details of grips. **(C)** Grips in use during surgery to attach a head plate. The cut-out in the head plate around the craniotomy provides room to attach an injury hub for a fluid percussion injury, however, a glass cover slip can be attached to this area for use as a cranial window, or it can be used for applying additional adhesive, if desired. For our experiments, a GRIN lens is attached to a round glass cover slip that is glued under the opening that is just rostral and to the left of the craniotomy. The lens is lowered into a separate craniotomy (not visible in this photo) as the head plate is lowered.

Prior to attaching the head plate, a thin layer of cyanoacrylate adhesive (Krazy Glue®) was applied to the skull (Yang et al., 2010; Crowe and Ellis-Davies, 2014). To position the head plate for implanting it was held by a set of two 3D-printed head plate grips (Figure 7C) mounted to the end of a steel probe holder (Figure 7B; Stoelting Co.) that was attached to a stereotaxic frame (Figure 7A). This tool was manipulated using the rostral-caudal and medial-lateral direction controls of the stereotaxic frame to guide the lens to the desire stereotaxic coordinates. The probe holder control was then lowered until the GRIN lens was touching the pial surface of the brain. After this, the assembly was lowered at a rate of 0.1 mm/min to the desired depth into the brain. Once the final depth was reached, cyanoacrylate glue was applied around the head plate and in any holes in the head plate that were not used for the window, GRIN lens, or other devices. For some animals, we left the large central hole open for later installation of an injury hub for inducing a traumatic brain injury using midline fluid percussion (Lifshitz et al., 2007). Stereotaxic coordinates will vary depending on the location of the brain region for imaging. Insertion depth is limited by the length of the GRIN lens and the angle of insertion.

It is important that the nose of the mouse is tilted upward for GRIN lens implants in rostral regions of the brain; otherwise the lens will not fully insert into the brain before the back of the head plate stops against the upper curve of the skull. To raise the nose so that the skull is more parallel with the head plate, the height of the bite bar on the stereotaxic frame can be increased. Likewise, for lateral implants the head should be tilted to the side to avoid having the head plate hit the upper curve of the skull and stop before the lens is fully inserted. For slightly lateral implants, this can often be accomplished by slowly adjusting one ear bar higher than another. However, adjusting an ear bar to access more lateral positions requires a larger angle and will result in the ear bar coming out of the ear canal. For these insertions, it will be necessary to adjust the angle of the stereotaxic arm that holds the probe holder (Note: Pressure should never be applied to one side of the head plate to force it to seal completely against the skull as this would cause the end of the GRIN lens to move rapidly in an arc through the brain damaging a much larger area of tissue than a proper, linear insertion path). When the lens is being implanted in one of these special areas, it is best to check the head angle or angle of the stereotaxic arm by lowering a head plate without a lens to the skull and adjusting the angles for implantation before attempting to implant a GRIN lens.

### Imaging

After acclimating to the room in their home cage for at least 30 min, mice should be anesthetized in an induction chamber under 3% isoflurane (Note: The heater for the water reservoir-pump system should be turned on about 20 min prior to inducing anesthesia). When vascular counterstaining is desired, a dye such as Texas Red-Dextran (Torres Filho et al., 2013) can be injected via a lateral tail vein (Levene et al., 2004). After anesthesia is induced, the mouse should be placed in the TRIO Platform. Light anesthesia can be maintained using 1% isoflurane.

Immediately prior to moving the mouse to the microscope, the coverslip should be cleaned with 70% isopropyl or ethyl alcohol. This can be done under a stereomicroscope or large, lighted magnifying glass. Once the window is cleaned, the TRIO Platform and compact anesthesia system are brought to the microscope. The head plate is positioned under the objective lens of the multiphoton microscope. A fiber optic light on a flexible metal shaft is useful for illuminating the head plate and glass window for positioning while using the brightfield setting on the microscope. For repeated imaging in mice with identical head plates, two to three guide bars can be fastened onto the microscope stage that will automatically position the platform for imaging. We have not noticed any movement of the platform over imaging sessions as long as 2 h. The platform could be taped into place if procedures are performed during the imaging session that might move the platform.

To test our system for *in vivo*, multiphoton imaging, we used GFAP-GFP mice (The Jackson Laboratory) with an implanted GRIN lens, as described in the surgical section. Aligning a GRIN lens for imaging was a more rigorous test of our system than aligning a cranial window. For acquiring *in vivo* images, we used a Vivo™ 2-Photon Microscopy Workstation (3i). To focus on the implanted GRIN lens, we used a 40X/0.6 NA air objective with a correction collar (Nikon), as previously described (Murray and Levene, 2012). GFP was excited using a Chameleon multiphoton laser (80 MHz, Coherent) tuned to 890 nm and emitted light was filtered using a 525/40 bandpass filter (Brightline®, Semrock, Inc.). For some imaging sessions, the vasculature was stained with Texas red-dextran dye (Life Technologies, Inc.) via a tail vein injection (Torres Filho et al., 2013). This dye was simultaneously excited with the 890-nm laser light used for exciting GFP to capture images in the green and red channels at the same time. A 612/69 nm bandpass filter (Brightline® filters by Semrock, Inc.) was used to filter the emission from the red dye. Scanning was controlled by Slidebook, a proprietary program that controls the microscope (3i, Inc.) and performs image analysis and processing. We used a 2 μs dwell time with pixel averaging (4/scan). Power at the sample was adjusted to ~35 mW using a Pockels cell (Conoptics, Danbury, CT, USA) which was controlled using a Slidebook interface.

## Results

### Summary of performance

The TRIO Platform *in vivo* integrated imaging support system (Figure 1) has been used over 40 times. The rigid base plate provided a sturdy, yet thin base for positioning and holding a mouse under a microscope objective. The elastomer covering over the nose cone provided increased collection of anesthesia, lowering the percentages of anesthetic to induce and to maintain anesthesia. It also greatly reduced or eliminated any odor of anesthesia in the room, which likely reduced exposure of researchers to the anesthetic. Additionally, the water channels remained clear and the base and water lines and connections remained water tight. Furthermore, the restraint system successfully positioned mice ranging from 25 to 55 g with little or no motion artifact from breathing or heartbeat. Moreover, the alignment was sufficient for the demanding technique of GRIN lens imaging in which the laser beam must be parallel to the axis of the lens to image deep brain tissue optimally.

### Characterizing the temperature distribution of the water-heated base plate

Temperature measurements of the top of the heated base plate were acquired from five equally-spaced positions in the middle of the assembled plate, starting near the nose cone where the head of the mouse is placed (Position 1), and ending in the section near where end of the tail rests on the plate (Position 5). A Fluke 62 Mini infrared thermometer (Fluke, Inc.) was used to make these measurements. Three sets of readings were acquired over two days. The first set was taken on Day 1; the second and third sets were acquired on Day 2. Prior to making measurements, the water in the reservoir-pump system (300 ml/min flow rate) was heated to 40°C, as measured with an immersible glass laboratory thermometer. The IR thermometer was checked by measuring the temperature of the water bath, which matched the temperature of the immersed thermometer. After this, the water inlet and outlet tubes were connected to the base plate via Luer lock fittings and then the circulating pump was turned on. Temperature readings were taken at 5-min intervals for the first 30 min and after this they were taken at 10 min intervals for a total of 60 min. On Day two the water was drained between Test 2 and Test 3 and the plate was allowed to return to room temperature (about 22°C). The water reservoir remained heated. For Test 2, the tubes were connected in reversed positions so that the water flowed in the opposite direction, as summarized below.

Test 1: Flow from nose cone to rear
Test 2: Flow from rear to nose cone
Test 3: Flow from nose cone to rear

A slight increase in temperature occurred over the first few time points before the temperature stabilized. Therefore, only the last six measurements (20–60 min.) were used to determine the mean temperature at each position (Table 1).

**Table 1:** **Mean temperature at top of base plate**.

	**Position number**	**Mean ± SEM (°C)**
	1	35.11 ± 0.55
	2	35.17 ± 0.59
	3	35.22 ± 0.60
	4	32.56 ± 0.78
	5	24.11 ± 0.22

*SEM is square error of the mean; n = 3 measurements for each position*.

Our heated base plate system used 8 m of 4 mm ID × 6 mm OD clear polymer tubing to make the delivery and return circuit (4 m each side) from the heated water reservoir to the platform and back to the reservoir. Some heat was lost as the water traveled through the tubing; thus, it was necessary to maintain the water in the reservoir at 40°C to maintain a desired temperature of 35°C on the top of the heated base plate. The length, wall thickness, diameter, and material composition of the tubing could affect the amount of heat loss, so future systems with different lengths and types of tubing may require a higher or lower reservoir temperature.

The rather large decrease in mean temperature recorded for Position five was independent of the direction of water flow. Thus, the decrease was most likely not related to the loss of temperature over the distance of the serpentine water channel in the plate. The silicone coating was thicker over the far end of the plate that included Position 5. This rubberlike, waterproof coating could serve as an insulator and is the most likely explanation for the lower temperature in this area. This did not affect the ability of the plate to warm a mouse because only the mouse's tail rests over Position 5.

### Inducing anesthesia

Anesthesia was induced using 3% isoflurane in an induction chamber connected to a SomnoSuite IDV gas anesthesia system (Damen et al., 2015). A mouse was considered anesthetized after it ceased attempting a righting response when turned on its back in the chamber. At this point the mouse was quickly transferred to the nose cone of the support system and isoflurane anesthesia was maintained at 1.5% for surgery and 1–1.5% for imaging (*n* > 40). We were able to keep the mouse fully anesthetized for over 2 h, the average length of our imaging sessions. Routine toe pinches every 15 min were performed to ensure that mice were anesthetized (National Research Council (U.S.). Committee for the Update of the Guide for the Care and Use of Laboratory Animals, 2011). Furthermore, by using the shroud over the nose cone and the activated charcoal scavenging system built into the SomnoSuite® system, we noticed that the smell of isoflurane was absent or greatly diminished when the shroud was in place. This is consistent with a recent report that predicts a much lower level of isoflurane gas escaping into the room (Damen et al., 2015).

### Fitting the platform into a small space

**Figure 8:**
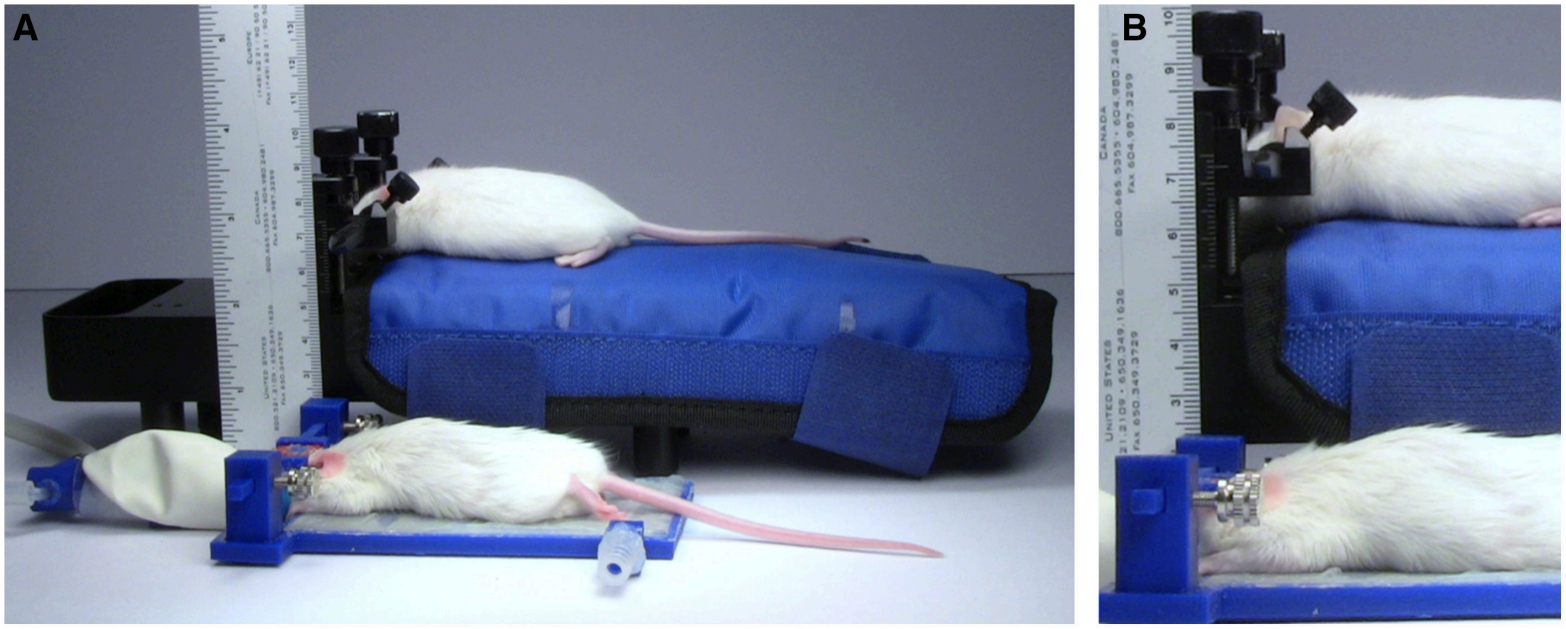
**Height comparison**. Comparison of the size of the TRIO Platform with a Mouse and Neonatal Rat Adaptor with an electrically heated warming pad. **(A)** Anesthetized mice restrained in the two systems. The TRIO system is capable of warming the entire mouse, but is still much smaller than the Mouse and Neonatal Rat Adaptor. **(B)** Close-up image of the two restraint systems. The top of the mouse head in the TRIO system is 2.2 cm above the table compared to 7.0 cm in the other system.

The TRIO platform fit under the relatively narrow space of 35 mm between our microscope objective lens and stage. Although the overall height of our system is 2.7 cm (Figure 8) from the bottom of the base plate to the top of the head plate holders, the top of the head plate is held at 2 cm. Thus the platform can be used in an imaging space only ~2.2 cm high, which means that it can be used on more microscope systems without replacing the microscope stage. In contrast, the Mouse and Neonatal Rat Adaptor (Stoelting Co.) was too tall to fit under the microscope objective (Figure 8). The distance to the top of the mouse head was about 75 mm, which would require removal or modification of the microscope stage. Similarly, a mouse placed on an electrically heated pad (RightTemp™ warming pad, Kent Scientific Corp.) or on a thermal pad (39DP Deltaphase Isothermal Pad, Braintree Scientific, Inc.) had a combined height that exceeded 35 mm. On the other hand, we could place the mouse under the objective lens on a thin, electrically heated plate (TCAT-2LV, Physitemp Instruments Inc.). However, the plate was wide and long and there was no good way to mount the head plate holder and a nose cone onto it in a secure manner.

### Suitability for *in vivo* microscopy

The support system has allowed us to image successfully multiple mice implanted with GRIN lenses over several weeks for a total of over 40 imaging sessions. The head plate and holder system held the head securely so that we observed little or no evidence of motion artifacts in our images. We also did not experience problems with misalignment of the lens with the laser beam.

**Figure 9:**
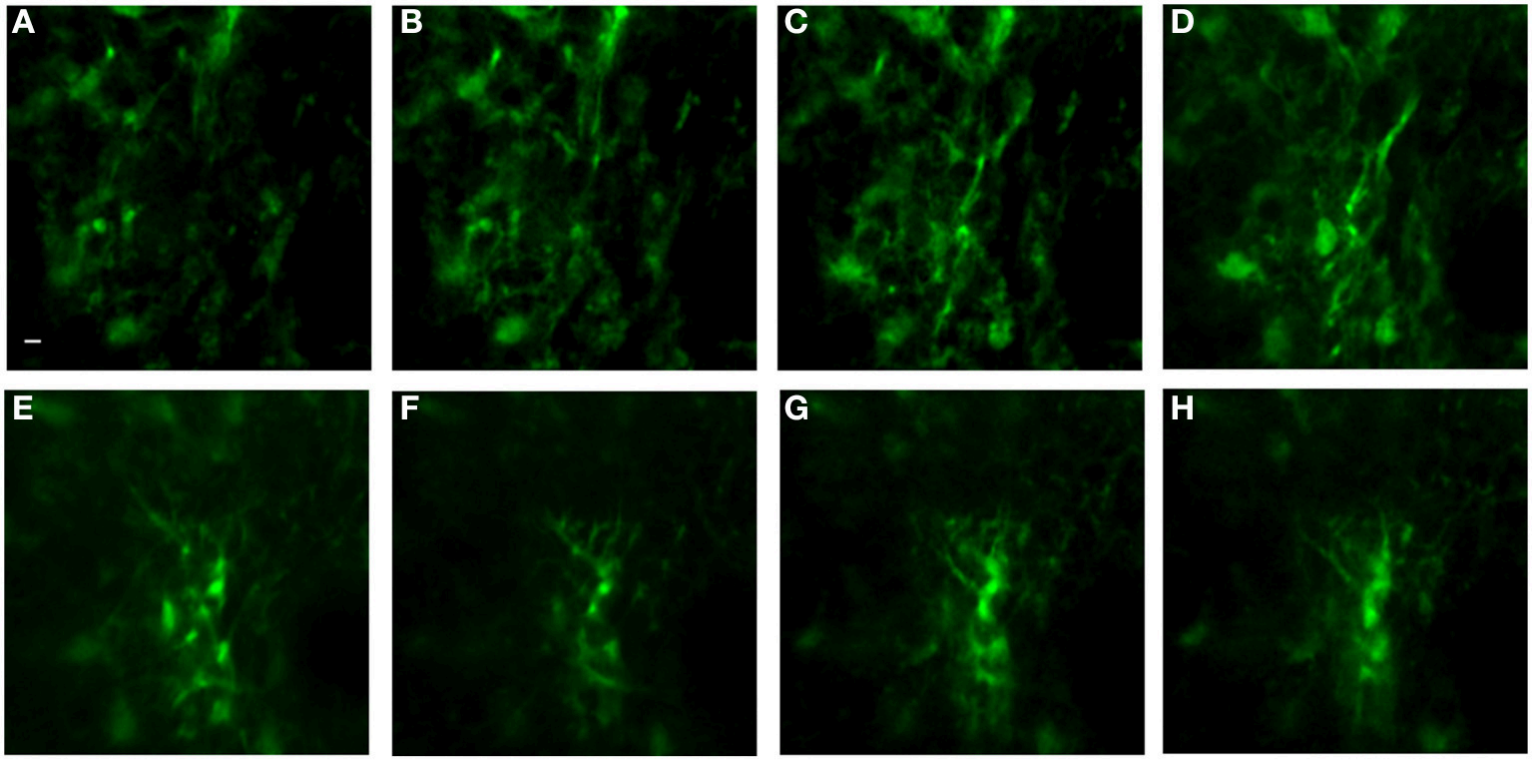
***In vivo* image set of astrocytes in lower levels of cortex using an implanted GRIN lens**. A z-stack of 51 images using 1 μm steps was acquired using a GRIN lens implanted in an adult GFAP-GFP mouse. Astrocyte cell bodies and processes were observed throughout the volume. Z-projections were made of several consecutive image planes (steps). All images were projections of five consecutive image planes with the exception of Image E in which 10 planes were used for the projection. The most dorsal image plane was defined as plane one and the most ventral image was plane 51. Z-projection planes for **(A–H)** were, as follows: **(A)** 1–5, **(B)** 5–10, **(C)** 10–15, **(D)** 15–20, **(E)** 25–35, **(F)** 35–40, **(G)** 40–45, **(H)** 45–50. A Gaussian filter with σ = 1.33 was applied to all channels to reduce background noise. Scale bar denotes 5 μm for all images.

**Figure 10:**
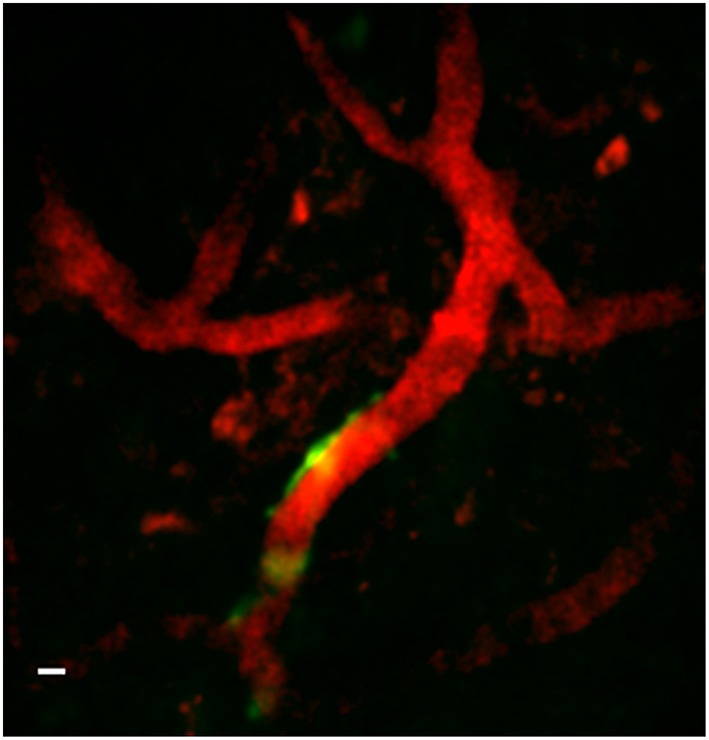
**Two-channel *in vivo* image using implanted GRIN lens**. Image is from prefrontal cortex of an adult GFAP-GFP mouse implanted with a 350-μm diameter GRIN lens. Texas red-dextran dye was injected into the tail vein to visualize the vasculature. A few glial cells expressing GFP under the GFAP promoter can be seen near one of a blood vessel near the center of the image. A Gaussian filter with σ = 1.33 was applied to all channels to reduce background noise. Scale bar denotes 5 μm.

Figure 9 is a representative set images of astrocytes in a 50-μm thick region of brain in a live GFAP-GFP mouse. The image set was taken through a 1.1-mm long, 350-μm diameter GRIN lens implanted into the prefrontal cortex. Cell bodies and processes can be seen at all levels through the 50-μm z-stack with some depths having more cell bodies that others. This type of imaging can virtually section the brain at varying depths and it can be repeated over time in the same animal. Figure 10 is a two-channel image showing blood vessels and GFP+ glial cells wrapped around a blood vessel.

### Summary of results

We have developed a low profile, multifunctional, *in vivo* brain imaging support system capable of positioning a mouse in precise alignment with an upright microscope, while simultaneously providing heat and anesthesia. The system is easily assembled and has a small footprint.

## Discussion

### Advantages of 3D printing

3D printing is a relatively inexpensive manufacturing process in both time and material costs. The method is also flexible, meaning it is typically only the matter of a dimensional change in a design program, such as SolidWorks®, to modify the prototype that we have built for supporting a rat or other laboratory animal. The head plate can be redesigned and repurposed if the region of interest is along the spine.

### Future construction methods and designs

In a few instances, a mouse's head plate became entangled in the overhanging bars that form the food hoppers in its cage. This resulted in head plates being dislodged. We have begun using a cage system without low overhanging bars to avoid this problem. Future head plate modifications will include reducing the size of the head plates so that they do not become caught between cage bars.

We have also determined, through measuring the mean temperatures of the base plate surface across the length of the plate that the silicone adhesive applied to seal the heated plate also acts as an insulator when applied in a sufficiently thick layer. The area with a relatively thick layer of silicone had a consistently lower temperature that was not dependent on the directions of warm water flow through the plate. This was not a problem for warming our mice because only the tail rested on this area. For future systems, a method to apply a uniformly thin coating of silicone (< 1 mm) should be developed.

### Potential impact

Currently, a variety of different components are combined to create a support system for *in vivo* optical imaging of anesthetized mice. Many of these systems include custom made components. Furthermore, some systems include the use of custom stages which may be due in part to the relatively large size of some components. Taken together, these conditions can make it difficult for other researchers to replicate these systems and make use of techniques that others have developed. We have combined the functions of head restraint, alignment, warming and anesthesia into one low-profile, compact system. Because of its small size, it should work on a wider range of confocal and multiphoton microscope systems than other larger systems. We hope that this system or one similar to it will provide a more standardized means of conducting these types of imaging experiments and thus enhance repeatability. Moreover, we hope that this system will enable more labs to conduct *in vivo* optical imaging experiments.

## Author contributions

Projected conceived by TM. VV and TM designed the platform system and BK contributed to refinements of the system, BK and KP performed temperature tests; VV and CD used the platform system for imaging experiments; and BK, VV, CD, KP, and TM wrote the manuscript and created figures. VV and BK contributed equally to this work.

## Funding

Funding for this work was provided by the National Institutes of Health (1R21HD075376 and 1R21NS090131).

### Conflict of interest statement

The authors declare that the research was conducted in the absence of any commercial or financial relationships that could be construed as a potential conflict of interest.

## Supplementary material

Design Files S1 **SolidWorks® design files for 3D printed components**.

Table S2 **Parts list for non-printed components**.
